# Role of the Neutrophil in the Pathogenesis of Advanced Cancer and Impaired Responsiveness to Therapy†

**DOI:** 10.3390/molecules25071618

**Published:** 2020-04-01

**Authors:** Bernardo L. Rapoport, Helen C. Steel, Annette J. Theron, Teresa Smit, Ronald Anderson

**Affiliations:** 1 Department of Immunology, Faculty of Health Sciences, University of Pretoria, Pretoria 0001, South Africa; helen.steel@up.ac.za (H.C.S.); atheron@up.ac.za (A.J.T.); ronald.anderson@up.ac.za (R.A.); 2 The Medical Oncology Centre of Rosebank, Johannesburg 2196, South Africa; teresasmit@mweb.co.za

**Keywords:** chemokines, CXC receptors 1 and 2, immunotherapy, granulocyte colony-stimulating factor, immune checkpoint inhibitors, myeloid-derived suppressor cells, reactive oxygen species, reparixin, SX-682, tumor-infiltrating lymphocytes

## Abstract

Notwithstanding the well-recognized involvement of chronic neutrophilic inflammation in the initiation phase of many types of epithelial cancers, a growing body of evidence has also implicated these cells in the pathogenesis of the later phases of cancer development, specifically progression and spread. In this setting, established tumors have a propensity to induce myelopoiesis and to recruit neutrophils to the tumor microenvironment (TME), where these cells undergo reprogramming and transitioning to myeloid-derived suppressor cells (MDSCs) with a pro-tumorigenic phenotype. In the TME, these MDSCs, via the production of a broad range of mediators, not only attenuate the anti-tumor activity of tumor-infiltrating lymphocytes, but also exclude these cells from the TME. Realization of the pro-tumorigenic activities of MDSCs of neutrophilic origin has resulted in the development of a range of adjunctive strategies targeting the recruitment of these cells and/or the harmful activities of their mediators of immunosuppression. Most of these are in the pre-clinical or very early clinical stages of evaluation. Notable exceptions, however, are several pharmacologic, allosteric inhibitors of neutrophil/MDSC CXCR1/2 receptors. These agents have entered late-stage clinical assessment as adjuncts to either chemotherapy or inhibitory immune checkpoint-targeted therapy in patients with various types of advanced malignancy. The current review updates the origins and identities of MDSCs of neutrophilic origin and their spectrum of immunosuppressive mediators, as well as current and pipeline MDSC-targeted strategies as potential adjuncts to cancer therapies. These sections are preceded by a consideration of the carcinogenic potential of neutrophils.

## 1. Introduction

The propensity of many types of epithelial cancers to develop at sites of chronic inflammation of both infective and non-infective origin is well-recognized, having been described by the distinguished German pathologist Rudolf Virchow more than 150 years ago [1]. Prominent examples of inflammation-related malignancies of infective and non-infective origin are summarized in Table 1 and Table 2, respectively. However, it was only many years later that insights into the pathophysiology of inflammation revealed the cellular and molecular mechanisms responsible for inflicting collateral damage on surrounding bystander tissues during hyperacute and chronic inflammatory reactions. Foremost among the cellular mediators identified were the phagocytic cells of the innate immune system, most prominently the abundant, highly mobile, reactive and destructive human blood neutrophil [2]. This cell, as well as several of its myeloid counterparts, such as monocytes and macrophages, generate substantial levels of toxic, reactive oxygen species (ROS), which, as well as being potentially mutagenic, also compromise the cellular mechanisms that repair oxidatively damaged DNA, thereby exacerbating carcinogenic potential.

Subsequent studies revealed that not only could neutrophils initiate carcinogenesis, but that their arsenal of indiscriminate toxic molecules could also drive the proliferation and spread of tumors [2]. Indeed, it is now realized that many types of established human tumors may even exploit neutrophils via production of neutrophil-recruiting and phenotype-reprogramming chemokines and cytokines, thereby co-opting these cells to disable anti-tumor host defenses in the tumor microenvironment (TME) [2].

The current review is focused primarily on: (i) the role of the neutrophil as a myeloid-derived suppressor cell (MDSC); (ii) MDSC-derived mediators, most prominently, but not limited to, ROS, which promote immunosuppression, resulting in tumor persistence, proliferation and spread; and (iii) targeting of neutrophil/MDSC-derived pro-tumorigenic mediators, as well as tumor-derived activators of these cells, as potential immunotherapeutic strategies in cancer. These sections are preceded by a consideration of the carcinogenic potential of neutrophils, as well as the interactions of these cells with established malignancies.

## 2. Pro-Oxidative, Pro-Carcinogenic Mechanisms of Neutrophils

Landmark studies communicated three decades ago clearly implicated the potential of the cell-permeant ROS, hydrogen peroxide (H_2_O_2_), acting in concert with intracellular ferrous iron, to inflict oxidative damage on the purine bases of DNA, particularly guanosine, via formation of hydroxyl radical [3,4,5,6]. These effects were evident following the exposure of isolated DNA, human blood lymphocytes, or cell lines to reagent H_2_O_2_, enzymatic H_2_O_2_-generating systems, or to activated phagocytes in vitro [3,4,5,6]. In the case of intact cells, oxidative damage to DNA was exacerbated by inactivation of several types of DNA-repair enzymes. These enzymes included: (i) poly (ADP-ribose) polymerase, a base excision repair enzyme that is oxidatively inactivated by another highly-reactive, cell-permeant, phagocyte-derived ROS, viz. hypochlorous acid (HOCl) [7]; (ii) the DNA glycolase OGG1, also involved in base excision repair, which is inactivated by phagocyte-derived nitric oxide [8]; and (iii) topoisomerase II, an enzyme that facilitates the excision of damaged DNA via strand scission/ligation, which is also inactivated by phagocyte-derived H_2_O_2_ [9]. These mechanisms underpin the oxidative damage inflicted on the DNA of bystander host cells at sites of inflammation, resulting in gene modifications, which precede cellular transformation [6], especially mutations that occur in tumor suppressor genes and oncogenes.

The existence of these pro-inflammatory/pro-oxidative mechanisms of carcinogenesis in the pathogenesis of inflammation-related human cancers is supported by observations that elevated systemic and urinary levels of 8-hydroxy-2-deoxyguanosine are significantly elevated in patients at risk for development of various types of cancer, including colorectal cancer [10,11].

## 3. Recruitment and Exploitation of Neutrophils by Tumors

Many types of tumor, as well as structural cells in the TME such as fibroblasts and mesenchymal stem cells, produce chemokines [12,13]. These, in turn, not only drive autocrine/paracrine proliferation of tumor cells that express chemokine receptors, but also promote the influx of neutrophils, most prominently those of the myeloid-derived suppressor cell (MDSC) phenotype. Chemokine-/chemokine-receptor-expressing cancers include those of the breast, colorectal origin, bladder, liver, pancreas, thyroid, oesophagus, stomach, cervix, ovary, lymphatics, squamous cell carcinomas of the head and neck and renal cell carcinoma [14,15,16,17,18,19,20,21,22,23,24]. Most pronounced among the neutrophil-recruiting/-activating chemokines produced by tumor cells and structural cells in the TME are CXCL5 (also known as epithelial neutrophil-activating peptide-78; ENA-78) and CXCL8 (interleukin-8; IL-8) [14,15,16,17,18,19,20,21,22,23,24]. These chemokines interact with the promiscuous chemokine receptor, CXCR2, expressed on neutrophils, as well as on many types of tumor cells, to initiate the influx of pro-tumoral neutrophils of the MDSC phenotype, while IL-8 interacts selectively with CXCR1 [12,13,15,16,17,19,22,23]. These tumor-associated neutrophils (TANs) have the potential to suppress the anti-tumor activities of tumor-specific infiltrating lymphocytes (TILs) of the CD4^+^ Th1 and cytotoxic CD8^+^ phenotypes, as well as driving angiogenesis and metastasis.

Increasing evidence has indicated that some types of established cancers may actually accelerate the process of myelopoiesis, resulting in moderate, or even profound, leukocytosis, characterized by increased numbers of immature myeloid cells in the bone marrow, blood and spleen [25,26,27,28,29]. In this setting, accelerated myelopoiesis appears to result from the production of bone marrow-stimulating growth factors by tumor cells, most notably the cytokines granulocyte colony-stimulating factor (G-CSF) and granulocyte/macrophage colony-stimulating factor (GM-CSF) [30,31,32,33,34,35]. In this context, it is noteworthy that an increased circulating neutrophil/lymphocyte ratio (NLR, defined as the absolute circulating neutrophil count divided by the lymphocyte count) is widely regarded as being a negative prognostic indicator in many, but not all, types of cancer reviewed in [36]. Other lines of evidence have strongly implicated neutrophilic infiltration of tumors as being predictive of adverse outcomes [36,37].

In this setting, a very recent clinical study reported by Kargl et al. is particularly relevant [38]. The study population recruited by these authors comprised three different cohorts of patients with non-small cell lung carcinoma (NSCLC; 73 patients undergoing lung resection; 28 receiving either programmed cell death-1 (PD-1)- or PD-ligand 1 (PD-L1)-targeted immune checkpoint inhibitor (ICI) immunotherapy; and 52 patients treated with second-line PD-1/PD-L1 therapy). The study, seemingly the first of its type in the clinical setting of cancer immunotherapy, described a previously unrecognized association between the extent of neutrophil infiltration and the exclusion of CD8^+^T cells from the TME [38]. On the basis of data derived from flow cytometric gene expression and multiplexed immunohistochemical analyses, the authors classified tumors into two major signatures. These were the “Active” and “Myeloid” signatures characterized by “robust” and “sparse” infiltration of CD8^+^ T cells, respectively, with neutrophils being highly associated with the “Myeloid” signature [38]. The two signatures were distinguishable according to the ratio of CD8^+^ T cells within the tumor mass and neutrophils within the stroma. Most importantly, this ratio was predictive of the response to PD-1-/PD-L1-targeted immunotherapy in the two treatment cohorts. The authors contend that, to their knowledge, “this is the first report that any myeloid lineage cell population contributes to ICI treatment failure” [38].

This latter contention is supported by the results of an additional experimental study undertaken by the same authors using a murine model of lung squamous cell carcinoma, a malignancy associated with significant neutrophilic infiltration [38]. In this study, animals were either untreated, or allocated to one of three different treatment groups: i) a PD-1-targeted monoclonal antibody, ii) a small molecule antagonist (SX-682) of neutrophil-mobilizing CXC receptors 1 and 2, or iii) both treatments. Only those animals in the combined treatment group demonstrated increased influx of CD8^+^ T cells and reduced tumor burden [38]. These findings are essentially in agreement with several earlier experimental studies in murine models of experimental colon cancer and NSCLC, in which neutrophilic inflammation resulting from tumor expression of the neutrophil-recruiting cytokine IL-17A was associated with disease progression and decreased responsiveness to PD-1-targeted immunotherapy [39,40]. In a somewhat similar context, another recent experimental animal study revealed that production of G-CSF by murine breast carcinomas was associated with attenuation of the therapeutic activity of breast tumor-targeted vaccines [41].

In addition, a study communicated very recently by Wisdom et al. has indicated that TANs may also attenuate the efficacy of traditional anti-cancer therapies such as radiation therapy [42]. In this retrospective, single-centre study conducted during the period 2006–2017, the authors investigated associations between pre-treatment circulating neutrophil counts and responsiveness to definitive chemo-radiation in patients (n = 278) with cervical cancer [42], a malignancy in which the TAN count had previously been reported to be an independent prognostic factor for short recurrence-free survival [43]. Wisdom et al. observed that lower pre-treatment circulating neutrophil counts were correlated with enhanced efficacy of chemo-radiation therapy (higher rates of local control, metastasis-free survival and overall survival) [42]. In an attempt to confirm their findings, the same authors used an autochthonous murine model of soft tissue sarcoma to investigate the effects of experimental depletion of neutrophils, using genetic and antibody-based strategies, on the efficacy of radiotherapy [42]. The authors observed that “neutrophil depletion prior to image-guided focal irradiation improved tumor response to radiotherapy”. They concluded that the efficacy of chemo-radiotherapy appears to be negatively impacted by the magnitude of the circulating neutrophil count (and presumably the extent of tumor infiltration by neutrophils), and recommended that pre-treatment measurement of the circulating neutrophil count represents an affordable, practicable biomarker of responsiveness to radiotherapy [42]. This contention is supported by the findings of a somewhat similar retrospective study undertaken by Cho et al. that covered the period 1986-2012 and encompassed 2456 patients with stage IA-IVA patients with uterine cervical cancer who had received “definitive radiotherapy with (37.4%) or without (62.6%) platinum-based chemotherapy” [44]. These authors compared the rates of locoregional-free (LFFS) and OS following categorisation of patients into two groups according to the presence or absence of tumor-related leukocytosis (TRL: >9000 leukocytes per ml blood) [44]. They observed that patients in the TRL^+^ group had significantly lower rates of complete remission, LFFS and OS than those in the TRL^-^ group [44].

Importantly, the American Society of Clinical Oncology (ASCO) Guidelines recommend vigilance when chemotherapy and radiation are used concurrently with G-CSF in lung cancer patients [45].

Although the aforementioned studies clearly underscore the existence of an ominous alliance between tumors and infiltrating MDSCs, predominantly neutrophils, in maintaining an immunosuppressive, therapy-unresponsive TME, considerable additional clinical research is clearly required to accurately identify and prioritize the best prognostic strategies. These include simply performing a circulating neutrophil count and/or measurement of the NLR, or, more definitively, in situ enumeration of TANs and TILs using computer-assisted imaging analysis procedures. Based on current, albeit somewhat limited data, the third option certainly seems the most promising in the case of solid tumors.

In this latter context, characterization of the densities of spatially positioned CD3 and CD8 cells in both the invasive margins and the center of the tumor have been shown to be associated with prognostic significance in patients with early colon cancer. The test yields a five-tiered classification (0–4), depending on the degree of CD3 and CD8 cell infiltration in the center of the tumor and the invasive margin. High levels of infiltration have been reported to be associated with improved outcomes in colorectal cancer patients [46,47]. The prognostic potential of this method has since been reported in other malignancies such as urothelial carcinoma of the bladder, resectable melanoma and cervical cancer [48,49,50], with studies in many other types of malignancy currently ongoing. Importantly, the application of this technology in cancer prognosis is undergoing continuous refinement with respect to the inclusion of other cell types, particularly immunosuppressive MDSCs and regulatory T cells (Tregs), immunosuppressive M2 macrophages, as well as expression of cytokine and immune checkpoint biomarkers of immunosuppression by tumor cells, immune cells and structural cells in the TME.

## 4. Myeloid-Derived Suppressor Cells (MDSCs)

Tumor-induced immature myeloid cells exhibit considerable heterogeneity and are generally not present in the circulation of healthy persons [36]. Also known as T cell-suppressive neutrophils, these tumor-co-opted MDSCs are predominantly of granulocytic (gMDSCs)/polymorphonuclear leukocyte (PMN-MDSCs) origin [36,51,52]. Although enriched in the so-called low-density neutrophil fraction, they have no recognized distinguishing markers of differentiation, essentially resembling immature neutrophils, both morphologically and phenotypically (CD11b^+^, CD14^−^, CD66b^+^, CD15^hi^) [36,51,52].

A very recent study by Aarts et al. has, however, reported that the identity of PMN-MDSCs may be somewhat less mysterious than originally thought [53]. These authors used a combination of discontinuous density centrifugation and magnetic cell sorting based on co-expression of CD11b and CD16 (expressed on mature neutrophils) to isolate immature and mature neutrophils from the bone marrow and blood of healthy adult humans, respectively [53]. They observed that suppression of T cell proliferation in five-day co-culture experiments was entirely dependent on the presence of mature, as opposed to immature, neutrophils and necessitated activation of these cells using stimuli such as the chemoattractant, N-formyl-L-methionyl-L-leucyl-L-phenylalanine (fMLP), bacterial lipopolysaccharide (LPS), or the cytokine tumor necrosis factor-α (TNF-α) [40]. ROS, as well as granule-derived factors, were the primary mediators of T cell suppression [53]. However, a potential criticism of this study by Aarts et al. [53] is the lack of inclusion of similar experiments using immature and mature neutrophils isolated from bone marrow and blood of patients with advanced cancer. Such a strategy would have controlled for possible differential immune suppressive activities of immature myeloid cells derived from healthy persons and cancer patients. Nevertheless, the study by Aarts et al. clearly underscores the T cell-suppressive potential of mature, circulating human neutrophils [53].

In a follow-up study, the same authors focused more closely on the mechanisms by which activated neutrophils isolated from the blood of healthy humans suppressed T cell reactivity [54]. In addition to confirming the involvement of CD11b-dependent release of toxic ROS and neutrophil granule constituents, they observed that T cell damage and dysfunction was associated with a process known as trogocytosis [54]. During trogocytosis, neutrophils acquire fragments of the cell membrane from adherent T cells, resulting in altered cell morphology, mitochondrial dysfunction and energy depletion. Importantly, and in distinction to their earlier study, the authors also investigated the numbers of low-density neutrophils, as well as spontaneous MDSC-like activity of neutrophils isolated from the blood of treatment-naïve, metastasis-free patients with either head and neck cancer (n = 8) or mammary cancer (n = 3) [54]. However, unlike the findings of several earlier studies, Aarts et al. were unable to detect either spontaneous MDSC activity of neutrophils from these cancer patients or increased numbers of low-density neutrophils. Indeed, acquisition of MDSC activity by neutrophils from cancer patients occurred in a manner similar to that observed with isolated neutrophils from healthy subjects [54].

Additional evidence, seemingly in support of the findings of Aarts et al. [53], originates from another recent study by Jimenez et al., who reported that isolated murine bone marrow progenitor cells acquired a MDSC-like phenotype associated with increased production of T cell-suppressive ROS following exposure to purified human C-reactive protein (CRP, 100 µg/ml) in vitro [55]. Moreover, treatment of isolated, presumably mature, human blood neutrophils with CRP effectively suppressed the proliferation of activated, co-cultured autologous T cells by a ROS-dependent mechanism [55]. However, the mechanisms by which exposure to CRP enabled both immature and mature neutrophils to acquire a MDSC-like phenotype was not established [55]. In this context, it is noteworthy that exposure of isolated human blood T cells to purified CRP (10 µg/ml), in the absence of autologous neutrophils, also appears to result in significant suppression of T cell activation and proliferation [56]. These observations seemingly strengthen the increasingly well-recognized link between chronic inflammation, elevated levels of circulating CRP and poor prognosis in patients with advanced cancer [57,58].

If substantiated in the clinical setting of advanced cancer in humans, the findings reported by Aarts et al. [53] may lend credence to the possible role played by TANs of the N1/N2 phenotypes in the pathophysiology of cancer. In this setting, neutrophils, like other cell types of the innate (M1/M2-like macrophages) and adaptive (Th1/Th2; Th1/Th17) immune systems, demonstrate plasticity of the phenotype, which appears to be determined by changes in the cytokine milieu of the TME [59]. The terminology “N1” versus “N2” TANs was proposed by Fridlender et al. in 2009, and is based on data derived from murine models of experimental tumorigenesis. These investigators reported on the key role played by the cytokine, transforming growth factor β1 (TGFβ1), present at high concentrations in highly immunosuppressive TMEs, in promoting the transition of the anti-tumor N1 TANs to pro-tumorigenic N2 TANs [59].

Although the concept of N1/N2 diversity of TANs has remained attractive [60,61,62], the evidence in support of the existence and roles of these putative cell types is largely circumstantial, with the precise identities of the cell types that function as MDSCs remaining largely unproven. Accordingly, we will continue to use the term MDSC in the remaining sections of this review.

## 5. Mechanisms by which MDSCs Promote an Immunosuppressive TME

MDSCs are present not only in the circulation and TME, but also in the secondary lymph nodes [63]. In the TME, these cells restrict the anti-tumor reactivity of TILs predominantly via pro-oxidative, as well as non-oxidative mechanisms, the latter mostly involving pre-packaged, granule-derived mechanisms [64].

### 5.1. Pro-Oxidative Mechanisms

Pro-tumorigenic MDSCs utilize an array of immunosuppressive strategies to disable the anti-tumor activities of TILs, most prominently, as mentioned above, the production of ROS and, to a lesser extent, reactive nitrogen species (RNS) [65]. Although neutrophils possess mitochondria, which also appear to be involved in the generation of ROS by MDSCs, the major contributor to the production of ROS by these cells is undoubtedly the membrane-associated, multi-component, electron-transporting, NADPH oxidase, also known as NOX2 [65]. As alluded to above, mechanisms by which MDSC-derived ROS suppress the reactivity of TILs in the TME appear to require intimate cell–cell contact mediated by the interaction of the β2-integrin CD11b/CD18(Mac-1), expressed on MDSCs with its receptor, intercellular adhesion molecule-1 (ICAM-1), expressed on TILs, ensuring the effective exposure of T-cells to MDSC-derived H_2_O_2_ [66,67].

Increased generation of ROS by MDSCs, resulting from up-regulation of NADPH oxidase activity, has been described by several groups of researchers using in vitro and in vivo models of experimental tumorigenesis, as well as in patients with cancer [68,69,70,71]. MDSCs isolated from both tumor-bearing mice and cancer patients demonstrated significant immunosuppressive effects on T cell proliferation and production of interferon-γ (IFNγ), while MDSCs from NADPH oxidase-deficient mice lost the ability to suppress T cell responses [68,70,71]. A further indication of the involvement of MDSC-derived ROS in suppression of T cell anti-tumor reactivity was the finding that the administration of agents that attenuated the production and/or reactivity of ROS completely abrogated the suppressive effects on T cells [71,72,73].

Notwithstanding induction of trogocytosis mentioned above [54], Treffers et al. have described additional, possibly related, mechanisms responsible for the induction of suppression of T cell proliferation by ROS, particularly H_2_O_2_ [63,74,75]. These include: (i) downregulation of expression of the signal-transducing ζ-chain of the T cell receptor for antigen, resulting in the loss of responsiveness of TILs to tumor antigens [76]; (ii) attenuation of activation of the transcription factor, nuclear factor-kappaB (NFκB), resulting in impaired cytokine production by T cells [77]; and iii) oxidation of the actin-remodeling protein, cofilin, which is involved in T cell activation and migration [63,78]. In addition, H_2_O_2_ has been reported to inhibit T cell chemotaxis initiated by the inflammatory chemokine, CXCL11, which is achieved via decreased expression of CXCR3 and failure of associated intracellular signaling mechanisms [79].

As well as producing ROS, MDSCs also produce RNS, such as nitric oxide (NO), via the activation of iNOS (inducible nitric oxide synthase) that can also suppress T cell function [80,81]. Powerful oxidizing agents, such as peroxynitrites, produced via the chemical reaction between NO and superoxide, are generated in high concentrations in areas where inflammatory cells and MDSCs accumulate in the TME. These high levels of peroxynitrites have been associated with tumor progression in many types of cancer [69]. Indeed, MDSC-derived peroxynitrites possess a range of immunosuppressive activities, including: i) induction of T cell apoptosis; ii) nitration of the T cell receptor (TCR) for antigens, resulting in the inability of these cells to bind MHC and respond to antigenic peptides [82,83]; iii) attenuation of recruitment of tumor-reactive T lymphocytes to the TME as a result of RNS-mediated nitration of chemokines such as CCL2 [84]; and iv) RNS-mediated interference with antigen presentation by dendritic cells [85].

These various mechanisms of immune dysfunction resulting from close contact of TILs with MDSC-derived ROS/RNS, some of which have been reviewed recently by Ohl and Tenbrock [65], are summarized in Table 3 with supporting references.

In addition to directly disabling the anti-tumor reactivity of TILs by pro-oxidative mechanisms, exposure to MDSC-derived peroxynitrite also renders tumor cells less sensitive to TILs [86]. In this setting, exposure to peroxynitrite results in structural modification of the HLA class I molecules present on tumor cells, attenuating the binding of antigenic peptides, causing a failure in the recognition of tumor-specific antigens by TILs [86].

### 5.2. Non-Oxidative Mechanisms

**Figure 1:**
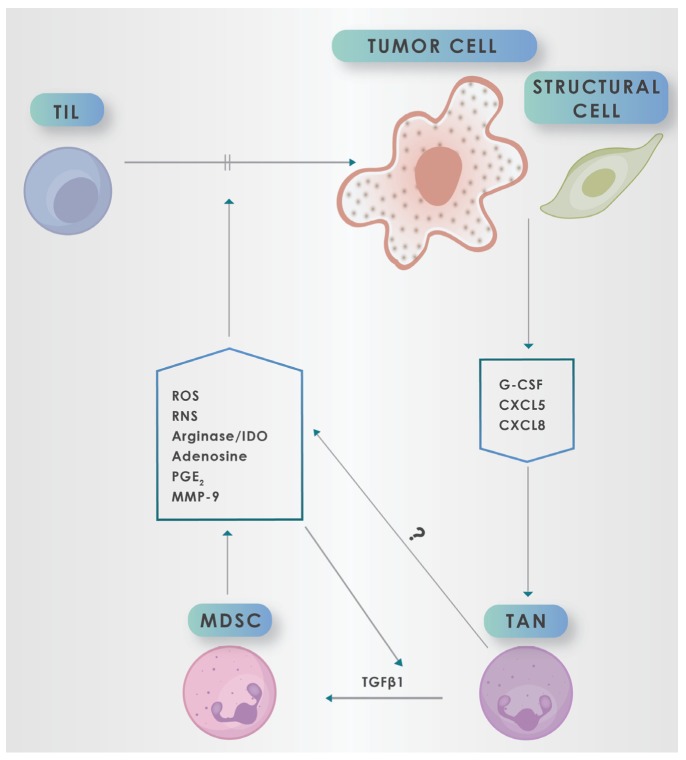
Tumor cells and structural cells promote migration of neutrophils into the tumor microenvironment (TME) via production of cytokines (G-CSF) and chemokines (CXCL5/CXCL8), which promote myelopoiesis and recruitment of neutrophils to the TME respectively, where they are known as tumor-associated neutrophils (TANs). In the TME, TANs, via transitioning to myeloid-derived suppressor cells (MDSCs) driven by exposure to proteolytically (MMP-9)-activated TGFβ1, release a range of potent mediators of immunosuppression. Prominent among these are reactive oxygen species (ROS), reactive nitrogen species (RNS), arginase, indoleamine-2,3-dioxygenase (IDO), adenosine, PGE2 and MMP-9. These mediators of immunosuppression target tumor-infiltrating lymphocytes (TILs), inhibiting (╫>) the anti-tumor reactivity of these cells.

Although ROS and, albeit to a lesser extent, RNS, appear to be the major mediators utilized by MDSCs to achieve T cell-targeted immunosuppression, these cells also produce a range of other immunosuppressive mediators, which augment the adverse activities of ROS/RNS [52,61,89,96,97,104,105]. These include mechanisms which restrict the availability of essential amino acids such as arginine, cysteine and tryptophan, recruitment of immunosuppressive regulatory Tregs, and production of immunosuppressive cytokines, specifically IL-10 and TGFß1, as well as prostaglandin E_2_ and adenosine [52,61,89,96,97,104,105]. These are summarized in Table 3, as well as in Figure 1.

#### 5.2.1. Amino Acid Deprivation

Arginase-1 released from the tertiary granules of MDSCs depletes the amino acid L-arginine, which is critical for T cell function, by converting it to L-ornithine and urea [63]. In addition, L-arginine is also a substrate for several other enzymes expressed by MDSCs including NOS, driving immunosuppression not only via depletion of arginine, but also through formation of NO [81]. Mechanisms of immunosuppression resulting from arginine depletion include: i) inhibition of T cell proliferation and production of IFNγ via downregulation of CD3ζ-chain expression [87]; and ii) arrest of the G0–G1 phase of the cell cycle as a result of impaired expression of cyclin D3 and cyclin-dependent kinase 4 in T cells [88]. In contrast to the aforementioned studies, a more recent study reported that arginase-1, which was expressed in MDSCs following exposure to activated T cells, was not, however, crucial for effective suppression of T cell reactivity [106]. Clearly, more studies are necessary to delineate the role of arginase-1 in the immunosuppressive activities of MDSCs.

Apart from arginine deprivation, MDSCs have also been shown to block T cell activation by: i) depletion of tryptophan through indoleamine 2,3-dioxgenase (IDO)-mediated mechanisms [96]; and ii) sequestration of cystine, thereby limiting the availability of cysteine, predisposing TILs to intracellular oxidative stress and cytotoxicity [97].

#### 5.2.2. Differentiation and Expansion of Tregs

MDSCs may also stimulate the generation of Tregs. These cells play an essential role in the maintenance of self-tolerance, but may also impede anti-tumor cell-mediated immune responses and promote cancer progression [90]. Although some disagreement is evident [107], several earlier studies have reported that MDSCs induce differentiation and/or proliferation of Foxp3+ Tregs in tumor-bearing mice [89,91,92] via mechanisms which involve the production of the immunosuppressive cytokines, IL-10 and TGFß1 [93,108]. In addition, others have reported that the monocytic subset of MDSCs has the ability to recruit Tregs to the tumor site in a CCR5-dependent manner, indicating that these cells may be most prominent with respect to the attraction of Tregs to the TME [94]. It therefore seems likely that an augmentative, immunosuppressive interplay between PMN- and monocytic-derived MDSC populations is operative in the TME.

It is also noteworthy that Tregs are less susceptible to oxidative stress-induced cell death compared to other T cell populations, which may further lead to selective enrichment of this cell type, thereby exacerbating immune dysfunction [95].

#### 5.2.3. Proteases

Neutrophil-derived proteases may also contribute to immunosuppression in the TME. In this context, the review by Treffers et al. [63] cited several early studies that showed that serine proteases cleave cytokines such as TNFα, IL-2 and IL-6, thereby impairing their functionality [63]. More recently, albeit in the setting of a murine model of colon cancer, neutrophil infiltration was most strongly associated with immunosuppression, which resulted from the release of matrix metalloproteinase 9 (MMP-9) and the resultant proteolytic activation of abundant latent TGFß in the TME [98]. With respect to clinical relevance, analysis of “two publicly available colorectal cancer gene expression datasets revealed that T cell signatures were lower in tumors with either high neutrophil or high TGFß signatures, but lowest when both neutrophil and TGFß signatures were high” [98]. Based on these findings, the authors proposed that T cells are excluded in TMEs in which neutrophils and TGFß are prevalent [98].

#### 5.2.4. Expression of Programmed Death Ligand-1 (PD-L1) and Fas ligand (FasL)

Tumor-infiltrating MDSCs may also induce immunosuppression via expression of high levels of PD-L1 [99]. This inhibitory ligand binds to its receptor, PD-1, suppressing T cell activation and promoting tumor escape and progression, while expression of Fas ligand (FasL) on PMN-MDSCs has been shown in pre-clinical studies to promote the apoptosis of TILs [100].

#### 5.2.5. Production of Adenosine and Prostaglandin E_2_

Other potential contributors to MDSC-mediated immunosuppression include the upregulated expression of the ectonucleotidases CD39 and CD73, resulting in the production of adenosine [81]. The interaction of adenosine with type A2_A_ adenosine receptors on CD8^+^ TILs results in the activation of adenylyl cyclase and the synthesis of immunosuppressive 3′,5′-cyclic adenosine monophosphate (cAMP) [101].

MDSCs, as well as tumor cells and other types of cells in the TME, also promote immunosuppression via production of prostaglandin (PG) E_2,_ which attenuates the anti-tumor activity of CD8^+^ TILs via interaction with adenylyl cyclase-coupled, PG-type E_2_ receptors on these cells [102].

#### 5.2.6. Other Mechanisms

An additional mechanism, albeit described in a murine model of tumorigenesis, by which systemic MDSCs interfere with distant anti-tumor immune mechanisms, is achieved via decreased expression of the lymph node homing receptor, L-selectin, on naïve T and B cells [109]. Although the precise mechanism of decreased expression of L-selectin is uncertain, it seems to involve MDSC-mediated cleavage of the adhesion molecule [109].

Other cell types of the innate and adaptive immune systems, which are susceptible to MDSC-mediated suppression of protective functions include natural killer (NK) cells and B cells, and this is also achieved via contact-dependent and -independent mechanisms involving ROS, NO, arginase and TGFβ1 [65,110].

## 6. Role of Neutrophils in Tumor Metastasis

In order for metastatic colonization to occur, tumor cells not only need to acquire a specific genetic profile that increases the likelihood of metastasis, but also to establish a pre-metastatic environment or ‘niche’ in distant organs [111]. In this context, and as alluded to earlier, a pre-metastatic environment is initiated by the release of tumor-derived G-CSF that mobilizes immature neutrophils from the bone marrow [112]. These newly released neutrophils move to the metastatic site before the tumor cells arrive, where they promote metastasis by inducing inflammation, tumor cell invasion and proliferation, remodeling of the extracellular matrix and suppression of the anti-tumor immune response. These events have recently been described in considerable detail in an extensive review by Leach et al., in which the involvement of neutrophils in promoting all stages of tumor progression and metastasis are highlighted, including the roles played by these cells in tumor cell intravascular survival, extravasation and promotion of metastatic growth [113]. Consequently, the present review focuses predominantly on the role of neutrophil extracellular trap (NET) formation in promoting metastasis

### 6.1. Role of Neutrophil Extracellular Traps (NETs) in Metastasis

G-CSF has also been linked, albeit in murine models of experimental tumorigenesis, to the formation of neutrophil extracellular traps (NETs) in both the intravascular and intra-tumoral environments [114]. NETs are formed when neutrophils externalize their nuclear DNA together with histones and antimicrobial granule proteins and proteases, such as neutrophil elastase and MMP-9, forming a web-like structure that traps, immobilizes and, in some cases, kills microbes, as well as some types of viral pathogens [115,116]. It is now becoming increasingly clear that NETs are formed during non-infectious inflammatory disorders, including cancer, contributing significantly to disease progression [117]. As such, the TME is an environment that is highly favorable to NETosis, with intra-tumoral NETs appearing to favor tumor growth [114,118]. Intravascular NETs can also contribute to metastasis via the capture of circulating tumor cells and delivery to distant organs [119]. In this context, Najmeh et al. have shown that β1-integrins, expressed on both tumor cells and NETs, mediate adhesion of circulating tumor cells to NETs [120]. In addition, interactions between β2-integrins on neutrophils and ICAM-1 on melanoma cells have been shown to anchor these cells to vascular endothelium, promoting extravasation [121]. Interestingly, Albrengues et al., using a murine model of experimental, tolerogenic, pulmonary tumor cell dormancy based on administration of breast cancer cell lines, observed that sustained lung inflammation caused by exposure to cigarette smoke or lipopolysaccharide triggered reactivation of dormant tumor cells [122]. This resulted from inflammation associated NETosis in which the proteases, MMP-9 and elastase expressed on NETs caused proteolytic remodeling of laminin, which, in turn, triggered the proliferation of dormant cancer cells via binding to the integrin α3ß1.

In addition, MMP-9 and other neutrophil proteases associated with intravascular NETs may increase vascular permeability and degradation of the extracellular matrix, allowing extravasation of cancer cells and metastasis [123,124]. Other reports suggest that NETs may contribute to metastasis via stimulation of tumor invasiveness and proliferation [114], while intra-tumoral NETs may restrict anti-tumor host defenses by impeding access of TILs to tumor cells [124]. The involvement of NETs in cancer progression is further supported by the reports of elevated levels of systemic citrullinated histone 3 (CitH3) found in individuals with advanced cancer [103]. Histone citrullination is catalyzed by high levels of peptidylarginine deiminase 4 (PAD4) expressed by neutrophils and is responsible for the chromatin decondensation associated with NETs [125]. Furthermore, a role for CitH3 as a prognostic marker of a poor clinical outcome has been suggested [126], while determining the expression of G-CSF by tumor cells may assist in identifying individuals who are at increased risk of developing metastasis driven by NETs [114].

### 6.2. Neutrophil-Derived Pro-Metastatic Cytokines, Growth factors and Granule Enzymes

MDSCs also produce cytokines such as hepatocyte growth factor (HGF) and oncostatin M (OSM), which are pro-metastatic via induction of an invasive phenotype and detachment of tumor cells, respectively [127]. Other MDSC-derived cytokines and proteinases which contribute to metastasis include vascular endothelial growth factor (VEGF), MMP-9 and elastase [52,61,105,128].

## 7. Adjunctive Therapeutic Targeting of MDSCs of Neutrophilic Origin

Strategies to attenuate the pro-tumorigenic activities of MDSCs are mostly based on monoclonal antibody (mAb) targeting of neutrophil-mobilizing cytokines, as well as pharmacological targeting of the chemokine receptors, CXCR1 and CXCR2. Other strategies include pharmacological targeting of MMP-9, IDO and receptor-mediated activation of adenylyl cyclase.

### 7.1. Cytokine Targeting

Targeting of the G-CSF/MDSC axis is an attractive option [35], which is attainable in the experimental setting via the administration of mAbs that neutralize G-CSF directly or block its receptor expressed on bone marrow precursor cells [129]. However, such a strategy is impractical in the clinical setting since it presents an unacceptably high risk of severe neutropenia and development of life-threatening microbial infection. Alternative strategies include the targeting of IL-17A or its receptor with mAbs such as secukinumab and brodalumab, respectively. IL-17A produced by Th17 cells promotes neutrophil activation and recruitment indirectly via receptor-activated production of IL-8 and G-CSF by various cell types, including structural cells [130]. In this context, the targeting of IL-17A is seemingly efficacious in the experimental immunotherapy of colorectal, breast and non-small cell lung cancers in murine models [40,131,132]. Although untested in the clinical setting of cancer chemotherapy, it is noteworthy that several members of the macrolide class of antibiotics such as azithromycin, clarithromycin and erythromycin attenuate the pro-inflammatory activities of neutrophils via inhibition of the production of IL-17A and IL-8 by both immune and structural cells [133,134]. Based on promising pre-clinical data, macrolide antibiotics may also have therapeutic potential in attenuating the pro-tumorigenic activity of MDSCs [135].

Based on the findings of Fridlender et al. [59], that TGFβ1 promotes the reprogramming of anti-tumor TANs of the N1 phenotype to N2 pro-tumorigenic TANs in the TME, targeting of this immunosuppressive cytokine using mAbs, drugs or other agents represents a potential strategy to counter the emergence of MDSCs. Such a strategy must, however, be weighed against the key role played by TGFβ1 in maintaining immune homeostasis. Alternatively, administration of recombinant type I interferons (IFNs), which have the opposite effect to TGFβ1 [59], may neutralize TGFβ1-mediated induction of N2 TANs. In this context, inducers of immunogenic cell death (ICD), which promote the release of endogenous type I IFNs from tumor cells in situ, may represent the most effective strategy [136,137].

### 7.2. Targeting CXCR1/CXCR2

Antagonists of CXC receptors, specifically dual antagonists of CXCR1 and CXCR2, have the potential to interfere with tumor-orchestrated recruitment of neutrophils/MDSCs into the TME. Exclusion of neutrophils/MDSCs from the TME may therefore improve the efficacy of chemotherapeutic and immunotherapeutic agents. Three CXCR1/2 antagonists, namely the small chemical entities, reparixin (formerly known as repertaxin) [138], SX-682 [38,139] and navarixin (MK-7123) [140] have recently entered the clinical arena of adjunctive therapy of cancer. All three of these agents are currently undergoing clinical evaluation in the setting of various types of advanced malignancy in combination with either immunotherapeutic or chemotherapeutic agents.

Reparixin is being evaluated in combination with paclitaxel in patients with triple-negative breast cancer, either newly diagnosed or relapsed following neoadjuvant chemotherapy, with paclitaxel alone as the comparator (NCT02001641; phase two trial). In this setting, reparixin may increase the efficacy of paclitaxel-driven ICD [141].

The other clinical trials involve the combination of a CXCR1/2 antagonist with a PD-1-targeted inhibitor (pembrolizumab). The first of these is an open-label, phase one/two trial in which patients with metastatic melanoma are treated with a combination of SX-682 and pembrolizumab (NTC03161431), while navarixin in combination with pembrolizumab is being evaluated in patients with selected, advanced metastatic solid tumors, specifically colorectal cancer, NSCLC and prostate adenocarcinoma (NTC03473925; phase two trial).

Although not directly related to the neutrophil-focused theme of the current review, another ongoing trial of this type is, nevertheless, noteworthy. This trial is focused on the efficacy of BMS-813160, an antagonist of CCR2/CCR5 involved in recruitment of monocytes/macrophages and Tregs to the TME. In this trial, the adjunctive anti-tumor potential of BMS-813160, used in combination with either chemotherapeutic agents or nivolumab (PD-1-targeted), is being evaluated in patients with advanced colorectal or pancreatic cancer (NTC03184870) [142].

### 7.3. Other Strategies

Although unproven in the clinical setting, other potential MDSC-targeted adjunctive strategies include: (i) mAb targeting of MMP-9 [143], which promotes activation of TGFβ1 in the TME [98]; (ii) small molecule inhibitors of MDSC-derived arginase-1 (CB-1158) [144] and IDO (indoximod) [145], which may preserve arginine and tryptophan, respectively, in the TME; (iii) attenuation of activation of adenylyl cyclase in TILs using antagonists of adenosine A2_A_ receptors (CPI-444 and AZD4635) and ATP ectonucleotidases [146], as well as inhibitors of cyclooxygenases; and iv) pharmacological targeting of PAD4, a key enzyme in NET formation. Additionally, recent data suggest that TRAIL-R2 (an agonistic antibody of the TNF-Related Apoptosis-Inducing Ligand Receptor 2) induces death of PMN-MDSCs in vitro and also potentiates the effect of the CTLA-4-targeted immune checkpoint blockade in animal models of experimental tumorigenesis [147].

Clearly, the clinical utility of all of the aforementioned MDSC-targeted, adjunctive anti-cancer therapies (summarized in Table 4) remains to be established, with CXCR1/2 antagonists seemingly the most promising.

## 8. Conclusions

A considerable body of evidence, much of it very recent, has underscored the ominous and effective tactics utilized by established tumors to intensify immunosuppression in the TME via enlistment and pro-tumorigenic reprogramming of neutrophils. These insights are of considerable potential value, enabling the identification of novel approaches for disease prognosis and therapy. In the case of the former, measurement of the circulating neutrophil count together with the NLR, possibly complemented by systemic levels of G-CSF, may represent an affordable prognostic strategy, which becomes particularly effective if augmented by quantitative, computer-associated imaging of immune cells in the TME. Although many MDSC-targeted adjunctive therapies are currently in development, the most promising and advanced of these involve targeting of the CXCR1/2 neutrophil/MDSC-mobilizing receptors.

## Figures and Tables

**Table 1:** Examples of inflammation-related malignancies of chronic infective origin. ^[2]^

Type of Malignancy	Associated Infective Agent
Squamous cell carcinoma of the bone, sinuses and skin	Chronic osteomyelitis most commonly caused by *Staphylococcus aureus*
Urinary bladder cancer	*Schistosoma haematobium*
Ovarian cancer	Pelvic inflammatory disease most commonly caused by *Chlamydia trachomatis* and *Neisseria gonorrhoeae*
Gastric cancer	Gastritis caused by *Helicobacter pylori*
MALT lymphoma	*Helicobacter pylori*
Lung carcinomas	Chronic and recurrent pulmonary infection as a result of various bacterial pathogens
Testicular cancer	Orchitis caused by mumps virus
Hepatocellular carcinoma	Hepatitis viruses B and C
Cervical cancer	Human papilloma virus
Kaposi’s sarcoma	Human herpes virus type 8

^[2]^ Reproduced with the approval of the authors: Anderson, Tintinger, Feldman. “Inflammation and cancer”, *S Afr J Sci*. **2014,** *110*, Art. #2013-0207, 6 pages. doi: 10.1590/sajs.2014/201130207. Published under a Creative Commons Attribution (CC-BY) license.

**Table 2:** Examples of inflammation-related malignancies of chronic non-infective origin. ^[2]^

Type of Malignancy	Associated Conditions
Colon carcinomas	Inflammatory bowel disease (Crohn’s disease, colitis)
Urinary bladder cancer	Long-term indwelling catheters, stones
Gall bladder cancer	Chronic cholecystitis, cholelithiasis
Oesophageal squamous cell carcinoma and adenocarcinoma	Chronic exposure to chemical irritants and acid reflux oesophagitis, respectively
Lung carcinomas	Cigarette smoking, pulmonary fibrosis, sarcoidosis
Mesothelioma	Asbestos inhalation
Head and neck cancer	Cigarette smoking
Skin cancer (basal cell/squamous cell carcinoma, melanoma)	Exposure to sunlight

^[2]^ Reproduced with the approval of the authors: Anderson, Tintinger, Feldman. “Inflammation and cancer”, *S Afr J Sci*. **2014,** *110*, Art. #2013-0207, 6 pages. doi: 10.1590/sajs.2014/201130207. Published under a Creative Commons Attribution (CC-BY) license.

**Table 3:** Pro-oxidative and non-oxidative mechanisms of myeloid-derived suppressor cell (MDSC)-mediated T cell dysfunction.

Mediators and Mechanisms of Pro-Oxidative Activity		
Mediator	Mechanism of Immunosuppression	Ref
**H_2_O_2_**	Activation of poly(ADP-ribose) polymerase resulting in depletion of nicotinamide adenine dinucleotide and adenosine-5′-triphosphate	[3]
**H_2_O_2_**	Trogocytosis	[54]
**H_2_O_2_**	Decreased expression of the TCR zeta-chain, resulting in decreased T cell activation and anti-tumor cytokine production, especially interferon-gamma	[68,70,71,74]
**H_2_O_2_**	Induction of T cell apoptosis	[75]
**H_2_O_2_**	Attenuation of activation of NFκB resulting in decreased production of T cell cytokines	[77]
**H_2_O_2_**	Oxidation of cofilin resulting in impaired T cell activation and recruitment	[63,78]
**H_2_O_2_**	Decreased expression of T cell CXCR3, resulting in failure of responsiveness to CXCL11	[79]
**Peroxynitrite**	Nitration of the TCR resulting in the failure to interact with MHC/antigenic peptides presented by APCs	[82]
**Peroxynitrite/RNS**	Induction of T cell apoptosis	[83]
**Peroxynitrite/RNS**	Nitrative inactivation of T cell chemokines such as CCL2	[84]
**Peroxynitrite/RNS**	Nitrative inhibition of antigen presentation by dendritic cells to T cells	[85]
**Non-Oxidative Mechanisms**		
**Arginase 1**	Depletes arginine necessary for many anti-tumor activities of T cells	[52,61,63,81,87,88]
**IL-10 and TGFβ1**	Differentiation and expansion of pro-tumorigenic of Foxp3^+^ regulatory T cells	[89,90,91,92,93,94,95]
**Indoleamine-2,3-dioxygenase**	Depletes tryptophan necessary for T cell proliferation	[96]
**Sequestration of cystine and cysteine**	Compromises T cell intracellular anti-oxidant defences	[97]
**Proteases**	Proteolytic inactivation of T cell-derived immunostimulatory cytokines such as IL-2, IL-6 and TNF-α; activation of latent TGFß1	[63,98]
**Expression of PD-L1**	Suppression of T cell activation via interaction with PD-1	[99]
**Expression of FasL**	Induces apoptosis of TILs	[100]
**Ectonucleotidases** **CD39 and CD73**	Promote formation of immunosuppressive adenosine	[101]
**Cyclooxygenases 1 and 2**	Promote formation of immunosuppressive PGE_2_	[102]
**Intra-tumoral NETs**	Impede access of TILs to tumor cells	[103]

**Table 4:** Potential MDSC-targeted adjunctive therapies of advanced malignancies.

Type of Adjunctive Therapy	Status	Ref
mAb targeting of G-CSF or its receptor	Seemingly impracticable	[35,129]
mAb targeting of IL-17A or its receptor	Promising pre-clinical findings in colorectal, breast and non-small cell lung cancers	[40,131,132]
Macrolide antibiotic targeting of IL-8 and IL-17A production	Uncertain	[135]
mAb targeting of TGFβ1	Promising, but poses the risk of dysregulated immune homeostasis	[59]
Administration of type I interferons to prevent N1→N2 TAN reprogramming	Uncertain, but administration of ICD-inducing strategies may be preferable	[59,137]
Administration of CXCR1/2 antagonists	Very promising, undergoing advanced clinical evaluation	[138,139,140,141]
mAb targeting of MMP-9	Unproven, but may represent an alternative strategy to prevent activation of latent TGFβ1 in the TME	[98]
Small molecule inhibitors of arginase-1 and IDO to preserve arginine and tryptophan, respectively, in the TME	Unproven	[144,145]
Small molecule antagonists of adenosine A_2A_ receptors, as well as inhibitors of ATP ectonucleotidases and cyclooxygenases to prevent activation of T cell adenylyl cyclase via production of adenosine and PGE_2,_ respectively	Unproven	[146]

Monoclonal antibody (mAb); indoleamine-2,3-dioxygenase (IDO).
